# Prescriptions

**Published:** 1895-12

**Authors:** 


### FOR COLDS.

When called in late stages of a cold, to relieve, spray the nares thoroughly with Dobell's or Seiler's solution and apply:

℞. Cocaine mur.... gr. x.
Menthol........ gr. xx.
Solid albolene.. ʒ ii.

Apply to nares on a small swab not oftener than every three hours. This gives immediate and delightful relief. Such treatment of colds attracts rather than repels patients, and gives immediate relief.—*Medical World.*

### TOOTHACHE.

℞. Acidi arseniosi......gr. ii.
Morph. Sulphatis....gr. i.
Creasoti.............q. s.

M. Ft pastam. Sig:—Apply by a bit of cotton-wool to carious portion. —*Prescription.*

### BRONCHITIS.

In cases of subacute or subchronic bronchitis, Dr. Eshner has prescribed of the following combination a teaspoonful every three hours:

℞. Ammonium chlorid.,

Sodium iodide........aa ʒ iii.

Syrup of tolu.

Syrup of senega.....aa ℥ iss.

If a spasmodic element be present, sodium iodide, two and one-half grains, may be added to each dose.—*Phil. Polyclinic.*

### FLATULENT COLIC.

℞. Tinct. nucis vomicæ..... ʒ i.

Acidi nitro muriaticidil. ʒ ii.

Spiritus chloroformi..... ʒ i.

Infus. gentianæ........ad ℥ vi.

M. Dose:—Tablespoonful three times daily after meals.—*Texas Medical Journal.*

### RINGWORM OF THE BEARD.

℞. Oil of cade.............. ʒi.
Red oxide of mercury..gr. xii.
Ammoniated mercury..gr. xlv.
Sublimed sulphur......gr. xx.
Simple cerate...... ...℥i.

Use this ointment morning and night.—*Quarterly Medical Journal.*

### RHEUMATISM.

In cases of acute rheumatism, Dr. Carpenter very often prescribes of the following combination, a teaspoonful every three hours:

℞. Sodium salicylate ........ʒiv.

Solution potassium citrate ʒiiss.

Syrup ginger............fl. ℥ss.

M. *Phil. Polyclinic.*

### GONORRHŒA.

An excellent—in fact, a beautiful —emulsion for gonorrhœa in the second stage, is the following:

℞. Balsam copaiba............℥j.
Milk of magnesia (Philips') ℥iij.
Aq. camph..............ad ℥viij.

M. Ft. Emulsion. Sig: Teaspoonful four times daily.—WM. R. LOWMAN IN *Medical Summary.*

### FURUNCULOSIS.

℞. Tinct. cinchonæ comp. fl. ℥ii.

Tinct Nucis Vomicæ fl.,

Kali chloratis............aa ʒi.

Aquæ.................q.s. ℥vi.

M. Sig.:—Tablespoonful every three hours.—*Medical Brief.*

